# Assessment of genetic diversity among wild rose in Morocco using ISSR and DAMD markers

**DOI:** 10.1186/s43141-022-00425-1

**Published:** 2022-11-01

**Authors:** Karim Saghir, Rabha Abdelwahd, Driss Iraqi, Nezha Lebkiri, Fatima Gaboun, Younes El Goumi, Maha Ibrahimi, Younes Abbas, Ghizlane Diria

**Affiliations:** 1 Biotechnology Unit, Regional Center of Agricultural Research of Rabat, National Institute of Agricultural Research (INRA), Rabat, Morocco; 2 Polyvalent team in R&D, Polydisciplinary Faculty, Sultan Moulay Slimane University, USMS, 23000 Beni Mellal, Morocco; 3 Polyvalent Team in R&D, Higher School of Technology of Fkih Ben-Salah, Sultan Moulay Slimane University, USMS, 23000 Beni Mellal, Morocco; 4 Laboratory of Plant, Animal and Agro-industry Productions, Faculty of Sciences, University of Ibn Tofail, Kenitra, Morocco

**Keywords:** Wild rose, Genetic diversity, ISSR, DAMD, Population structure

## Abstract

**Background:**

Morocco is considered one of the main biodiversity hotspots in the Mediterranean region and contains various plant species including wild and domestic Rosa. This genus is the most important among cultivated ornamental plants in the world, with a high economic value in cosmetics, pharmaceutical industries, and floriculture. In the present study, genetic diversity among the collected accessions of wild *Rosa* species in Morocco was assessed using Inter-Simple Sequence Repeat (ISSR) and Directed Amplification of Minisatellites DNA (DAMD) markers.

**Results:**

Results confirmed that both markers used have a good efficiency to assess genetic diversity in wild roses. Ten ISSR and eight DAMD primers amplified 276 and 203 loci, with an average of 27.4 and 25 polymorphic alleles per primer, respectively. The polymorphic information content (PIC) values were 0.34 with ISSR and 0.31 with DAMD. Analysis of molecular variance (AMOVA) showed that genetic variation in wild rose occurs mainly within populations (86%) rather than between populations (14%). The region of Azrou (Middle Atlas of Morocco) is the area that registered the highest genetic diversity in the present study with He = 0.21. The 39 rose accessions were divided into three main groups with among-group similarity of 30%. Principal component analysis and the hierarchical classification were consistent with genetic relationships derived by structure analysis.

**Conclusion:**

The findings revealed that the patterns of grouping are weakly correlated with geographical origin. ISSR and DAMD markers showed that the accessions have a good genetic diversity.

## Background

The genus *Rosa* represents one of the largest genera in the Rosaceae family and is comprised of more than 200 terrestrial species and more than 18,000 cultivars but only ten species have contributed to modern commercial roses [1]. Wild rose has extremely high economic, ornamental [2, 3], and medicinal values [4]. Rose cultivars are derived from eight to ten wild species hybridization [5]. Several researches reported that *Rosa* distribution concern the temperate and sub-tropical regions of the northern hemisphere, specifically in Europe, Asia, the Middle East, and North America [6, 7]. However in North Africa, particularly in Morocco, fifteen species of wild roses, which spread in the northern half of the country, particularly in the Atlas Mountains and the Rif, were reported by Fennane et al. (1999) [8].

Recent research has classified the genus *Rosa* into four subgenera: *Hulthemia*, *Platyrhodon*, *Hesperhodos*, which are monotypic or include two species, and *Rosa* genus, which contains all the remaining species, grouped into 10 sections [9]. Generally, the classification of the *Rosa* genus has posed many problems to taxonomists as a gradation between many subdivisions is slight, and in many instances, segregation is a matter of opinion [10]. Genetic relationships within this genus are confusing due to the variability of species and the weak barriers to intraspecific hybridization [11]. For this purpose, it is recommended to identify the species and the biological relationships within the *Rosa* genus for use in genetic improvement [12, 13]. Molecular markers were found to be more efficient in selection compared to conventional morphology-based methods. They provide easy and accurate access to genetic variability and determine polymorphism at the DNA level without environmental interference [14].

Since the mid-1980s, genome identification and selection has progressed rapidly with the help of PCR technology. A large number of marker protocols that are rapid and require only small quantities of DNA have been developed [15]. The widely-used PCR-based markers are RAPDs [16], SSRs or microsatellites [17], AFLPs [18], ISSRs [19], and DAMDs [20].

In the 1990s, molecular markers were developed for rose cultivar identification [21, 22] and several of these were tested to identify species relationships in Rosa [21, 22]. used RAPDs to examine the relationships among cultivars and a limited number of wild species. The work of [21] showed a distinction between a group of cultivars and that of wild species. But since 1994, a new molecular marker technique called inter simple sequence repeat (ISSR) has been available [19]. ISSR-PCR is a technique that originated from microsatellite regions and uses microsatellite sequences as primers (16–18 bp) in a polymerase chain reaction to generate multi-locus markers [23]. DAMD, as well, has been developed on the basis of preserved gene regions and can easily generate functional markers related to a given plant phenotype [24]. Both molecular markers are generated from the microsatellite-rich portions of the genome [25].

ISSR and DAMD markers are becoming important tools for genetic diversity analysis in plants. They collectively provide a comprehensive description of the nature and the extent of diversity, its genetic relationship, and germplasm management [26–31].

In Morocco, studies on wild roses are very rare even absent at morphological, anatomical, chemical, or genetic levels. The only studies provided to date concern morphological aspects [8]. To fill this gap, particular attention has been devoted to the genetic diversity of wild roses in order to know the potential of this natural resource as a natural heritage, to understand the species relationships with the aim of preservation and proposition of a reliable genetic improvement program in the context of current climate change.

A study of polymorphism among wild rose accessions was carried out using ISSR and DAMD markers. Therefore, the aim of this study is to characterize the genetic variability of wild rose species available in Morocco and the relationship between wild genetic pools. This will allow of performing breeding programs.

## Methods

### Collection of plant materials

**Fig. 1:**
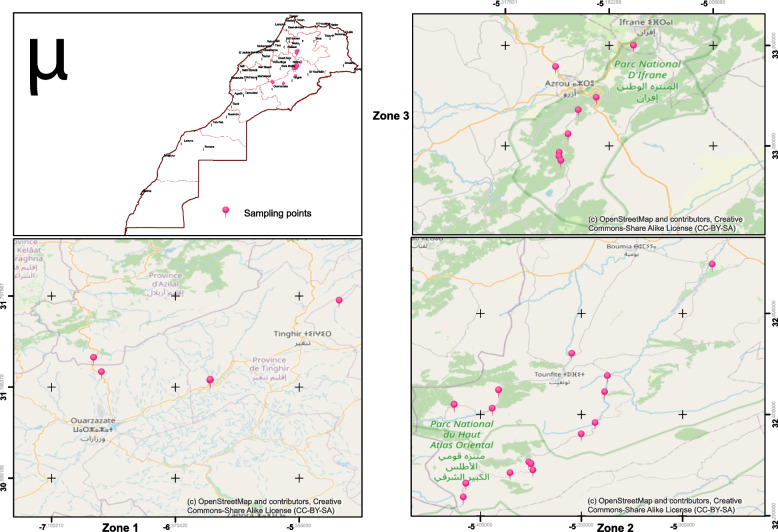
Map of Morocco illustrating the geographic distribution of the 39 Moroccan Rose accessions. Zoned 1: South East, zoned 2: High Atlas, and zoned 3: Middle Atlas

Thirty-nine accessions of *Rosa* sp. including 4 accessions of hybrid rose (3 *Rosa damascena* (KMG1, KMG3, and KMG4) and unknown (TOGH)) were collected from different geographical provinces of Morocco (Ifrane, Midelt, Azilal, Errachidia, Tinghir, and Ouarzazate) ranging from 1260 to 2358 m altitude (Fig. 1, Table 1). The sampling mode used in this study is simple random.

**Table 1:** List of Moroccan rose accessions used for molecular analysis

Site	Code	Accession	No.	Altitude (m)
**Azrou**	AZR1	Wild	1	1260.31
	AZR2	Wild	2	1802.00
	AZR3	Wild	3	1813.00
	AZR4	Wild	4	1882.29
	AZR5	Wild	5	1859.75
	AZR6	Wild	6	1868.00
	AZR7	Wild	7	1919.00
	AZR8	Wild	8	1783.22
**Tounfite**	ABA	Wild	27	1563.25
	TOGH	Hybrid	28	1869.40
	TOGS	Wild	38	1869.40
	MEG	Wild	39	1839.00
**Bouadel**	BOA1	Wild	33	2000.84
	BOA2	Wild	23	2027.07
	BOA3	Wild	34	1875.67
	BOA4	Wild	35	1783.80
	BOA5	Wild	36	1777.00
	BOA6	Wild	29	1774.06
	BOA7	Wild	37	1770.50
**Tamalout**	TAM1	Wild	22	2237.93
	TAM2	Wild	21	2261.00
	TAM3	Wild	10	2267.24
**Akka Ait Ouyed**	ANO1	Wild	31	2311.00
	ANO2	Wild	11	2215.60
**Agoudim**	PAG1	Wild	25	1916.00
	PAG2	Wild	24	1916.00
	AGD1	Wild	26	2070.57
	AGD2	Wild	20	2184.50
**Tirghist**	AEG1	Wild	32	2143.50
	AEG2	Wild	30	2136.24
	AEG3	Wild	18	2358.00
**Taoudaat**	TAD1	Wild	19	1447.69
	TAD2	Wild	14	1447.69
**Kela Mgona**	KMG1	Hybrid	16	1453.25
	KMG2	Wild	17	1450.00
	KMG3	Hybrid	13	1459.50
	KMG4	Hybrid	15	1458.29
**Ait Touda**	IGN	Wild	12	1761.67
	TIG	Wild	9	1975.64

### DNA extraction and purification

Fresh young leaves (50 mg) were freeze-dried. Total genomic DNA was extracted from the young leaves using the CTAB method [32]. The leaves were crushed using 1 ml of preheated extraction buffer [PVP 0.2%, Tris-HCl 1 M pH 8.0, NaCl 5 M, 2% CTAB, EDTA 0.5 M, 0.2% β-mercaptoethanol] and incubated for 1 hour at 65°C. Then they were treated with an equal volume of chloroform mixture:isoamylic alcohol (24:1; v/v). Tubes were centrifuged at 13,000 rpm at 4°C for 15 min. After centrifugation, the aqueous phase obtained was transferred to a new tube and 700 μl cold isopropanol was added. DNA was precipitated by centrifuging at 13,000 rpm at 4°C for 10 min. Resulting pellets were washed twice with 70% ethanol. Isolated DNA was air-dried and dissolved in 200 μl of TE buffer. DNA quality was evaluated by spectrophotometry and visualized under UV light after electrophoresis on 1% agarose gel.

### ISSR analysis

All 39 accessions were analyzed with 10 ISSR primers (Table 2). DNA amplification was performed in 10 μl per reaction. PCR reaction contained 25 ng of genomic DNA, 2.5 μl of 1X buffer (dNTP, MgCl_2_), 0.5 U of TaqDNA polymerase (BIOLINE, London, UK), 0.5 μM of each forward and reverse primers and 5.3 μl of pure water. Amplification was carried out in thermocycler (Aeris^TM^) with the following program: an initial denaturing step of 5 min at 95 °C, followed by 35 cycles, each had a 95 °C denaturing step for 30 s, a hybridization at 52 to 58.5 °C (depending on primers) for 1 min and extension step at 72 °C for 2 min. The final elongation step was set to 72°C for 8 min. The amplification products were stored at 4 °C until visualization using polyacrylamide gel electrophoresis. After electrophoresis, the gel was removed from the plates and stained with ethidium bromide solution for 5 min. A 50-bp ladder (BIOLINE) was used to evaluate the approximate molecular weight of amplification products.

**Table 2:** Primers selected for ISSR analyses in *Rosa* sp.

ISSR primers	Sequence (5′-3′)	Ta (°C)
I1	GAGAGAGAGAGAGAGAGAC	52
I3	GAGAGAGAGAGAGAGAGAA	52
UBC811	GAGAGAGAGAGAGAGAC	52
UBC834	AGAGAGAGAGAGAGAGCT	52
UBC841	GAGAGAGAGAGAGAGACTC	52
UBC842	GAGAGAGAGAGAGAGACG	52
UBC880	GGAGAGGAGAGGAGA	58.5
UBC889	DBDACACACACACACAC	52
ISSR2	GAGAGAGAGAGAGAGAC	50
ISSR15	GTGGTGGTGGC	53

A, T, C, and G: nitrogenous bases; *Ta* temperature annealing; B: C, G, or T; D: A, G, or T

### DAMD analysis

Using eight DAMD primers (Table 3), all 39 accessions were analyzed. DNA amplification was carried out in 10 μl per reaction. PCR reactions contained 25 ng of genomic DNA, 2.5 μl of 1X buffer (dNTP, MgCl_2_), 0.5 U of TaqDNA polymerase (BIOLINE, London, UK), 0.5 μM of each forward and reverse primers and 5.3 μl of pure water. Amplification was performed in thermocycler (Aeris^TM^) with the following program: an initial denaturing step of 3 min at 94°C, followed by 35 cycles, each had a 92°C denaturing step for 45 s, a hybridization at 40 to 70°C (depending on primers) for 2 min and extension step at 72°C for 2 min. The final elongation step was set to 72°C for 5 min. The amplification products were stored at 4°C until visualization using polyacrylamide gel electrophoresis.

**Table 3:** Primers selected for DAMD analyses in *Rosa* sp.

DAMD primers	Sequence (5′-3′)	Ta (°C)
HBV3	GGTGAAGCACAGGTG	50
HBV5	GGTGTAGAGAGGGGT	49
HVR	CCTCCTCCCTCCT	40
INS	ACAGGGGTGGGG	40
M13	GAGGGTGGCGGCTCT	60
URP2R	CCCAGCAACTGATCGCACAC	70
URP9F	ATGTGTGCGATCAGTTGCTG	60
URP25F	GATGTGTTCTTGGAGCCTGT	59

A, T, C, and G: nitrogenous bases; *Ta* temperature annealing

### Statistical analysis

#### Genetic diversity and frequency analysis

ISSR and DAMD profiles were scored as present (1) or absent (0) for each entry and a binary qualitative data matrix was constructed. Very faint and non-reproducible bands were omitted from scoring. The genetic parameters such as observed number of alleles (na), effective number of alleles (ne) [33], Shannon's Information index (I) [34], expected heterozygosity (He), polymorphic band (PB), and percent of polymorphic band (PPB) were calculated by GenALEx program version 6.5. Analysis of molecular variance was calculated by SAS software version 9. Principal component analysis (PCoA) for the 39 accessions of roses was generated using GENSTAT 18th edition.

#### Marker efficiency analysis

The performance of the molecular markers was measured by calculating various parameters including polymorphism information content (PIC), effective multiplex ratio (EMR), marker index (MI), and resolving power (Rp) for each primer by program iMEC (https://irscope.shinyapps.io/iMEC/).

#### Genetic relationship analysis

Clustering was done to determine the genetic distance between individuals and to verify the consistency of population genetic variation. The dissimilarity index was performed for cluster analysis using WNJ (Weighted Neighbor-Joining) method in DARWIN program version 6.0.4. The bootstrap analysis running 1000 replication was employed to determine a sampling variance of the genetic similarities calculated from the data sets gained of different marker systems [35].

#### Genetic structure

The Bayesian-based model carried out the genetic makeup of rose accessions. This was analyzed by STRUCTURE (version 2.3.4) and was compressed into a single “Zip-Rar” file and then uploaded online to “Structure Harvester” (http://taylor0.biology.ucla.edu/structureHarvester/).

## Results

**Fig. 2:**
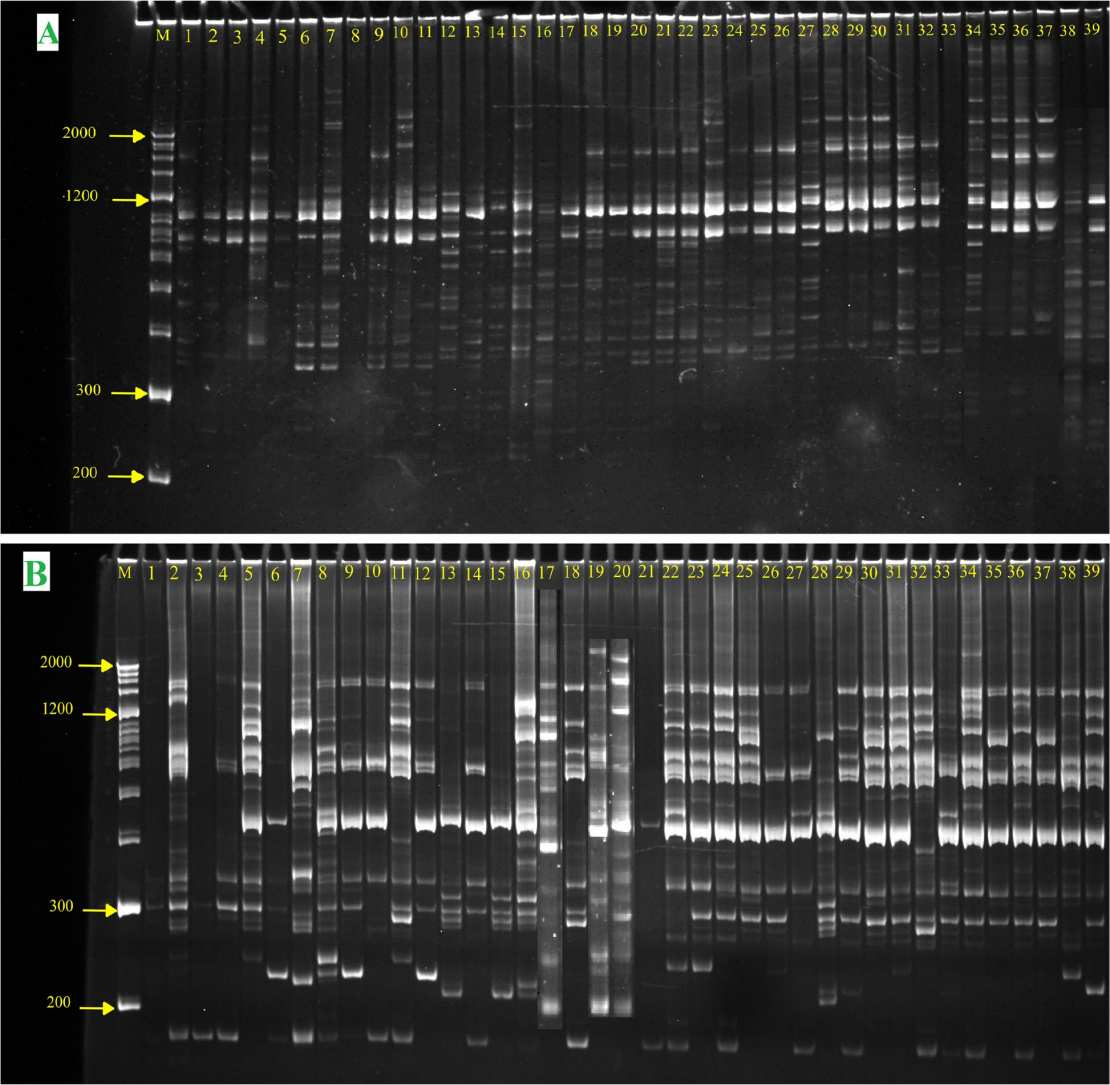
Amplification profile of M13 primer (**A**) and UBC841 (**B**) among 39 Rose accessions; M, DNA ladder

A total of 13 ISSRs and 10 DAMDs were used. Ten ISSR primers (I1, I3, UBC811, UBC834, UBC841, UBC842, UBC880, UBC889, ISSR2, and ISSR15) and eight DAMD primers (HBV3, HBV5, HVR, INS, M13, URP2R, URP9F, and URP25F) were selected as they gave better amplification products and showed clear polymorphism (Fig. 2).

### Polymorphism and efficiency of ISSR markers

ISSR primers produced 276 fragments among which 274 (99.27%) were polymorphic. A set of 10 ISSR primers detected polymorphic bands that varied from 13 (ISSR2) to 39 (UBC842) with an average of 27.4 bands per primer. Altogether, eight primers (I1, I3, UBC811, UBC841, UBC842, UBC880, UBC889, and ISSR2) presented 100% of polymorphic rates, while the lowest polymorphic percent (94.11%) was detected at UBC834. The highest PIC value was recorded for ISSR2 (0.37) followed by ISSR15 (0.36) while the lowest (0.29) was for UBC842 with an average of PIC of 0.34. Effective multiplex ratio (EMR) spanned from 3.9 (UBC834) to 12.1 (ISSR15) with 9.4 as the average. Marker index (MI) was calculated to recognize the usefulness of the ISSR primers system on *Rosa* sp. which maximum was for ISSR15 (4.42) followed by UBC811 (4.15) while the lowest for UBC834 (1.17) along with a mean of 3.05 per primer. Resolving power average value (Rp) was 11.95. UBC842 primer counted the highest value (16.92) while UBC834 showed the lowest value (5.74) (Table 4).

**Table 4:** Efficacy of primer polymorphism calculated with iMEC in *Rosa* sp. accessions using ISSR and DAMD primers

Marker Type	Primers	PIC	EMR	MI	Rp	TSB	PB	PPB
**DAMD**	HBV3	0.357	4.769	1.702	5.8462	13	13	100
	HBV5	0.343	11.128	3.816	14.2051	34	34	100
	HVR	0.3	7.538	2.261	14.6154	31	31	100
	INS	0.288	5.846	1.683	9.5897	26	26	100
	M13	0.315	11.769	3.707	16.1538	44	44	100
	URP2R	0.374	2.769	1.035	2.6154	9	6	66.66
	URP9F	0.303	4.949	1.499	6.4103	20	20	100
	URP25F	0.251	4.667	1.171	9.3333	26	26	100
	*Mean*	*0.316*	*6.679*	*2.109*	*9.846*	*25.37*	*25*	*95.83*
**ISSR**	I1	0.342	9.718	3.323	15.3333	30	30	100
	I3	0.342	9.385	3.209	13.1795	29	29	100
	ISSR2	0.374	6.821	2.551	6.8205	13	13	100
	ISSR15	0.365	12.103	4.417	14.6154	31	30	96.77
	UBC811	0.356	11.667	4.153	13.7436	32	32	100
	UBC834	0.301	3.897	1.172	5.7436	17	16	94.12
	UBC841	0.34	10.513	3.574	13.9487	33	33	100
	UBC842	0.291	8.949	2.604	16.9231	39	39	100
	UBC880	0.312	6.821	2.128	8.1026	26	26	100
	UBC889	0.357	9.59	3.423	11.1282	26	26	100
	*Mean*	*0.338*	*8.946*	*3.055*	*11.953*	*27.6*	*27.4*	*99.08*

*PIC* polymorphism information content, *EMR* effective multiplex ratio, *MI* marker index, *Rp* resolving power, *TSB* total scored band, *PB* polymorphic bands, *PPB* percent of polymorphic band

### Polymorphism and efficiency of DAMD primers

Two hundred and three fragments were generated by DAMD primers among which 200 (98.52%) were polymorphic. A set of eight DAMD primers detected polymorphic bands which varied from 6 (URP2R) to 44 (M13) with an average of 25 bands per primer. However, all primers generated multiple patterns ranging from 9 to 44 with an average of 25.37 alleles per loci. Absolutely, seven primers (HBV3, HBV5, HVR, INS, M13, URP9F, and URP25F) registered 100% of polymorphic rates, while the polymorphic percent observed was as low as 66.66% for URP2R. URP2R recorded the highest PIC value of 0.374 followed by HBV3 (0.36) whereas the lowest was URP25F (0.25) with a mean of PIC of 0.31. Effective multiplex ratio (EMR) spanned from 2.77 (URP2R) to 11.77 (M13) with an average of 7.9 per primer. The marker index (MI) was maximum (3.82) for HBV5 followed by M13 with 3.71, while the lowest was 1.03 for URP2R with a mean of 2.11. The highest value counted for resolving power (Rp) is 16.15 for M13 while the lowest was 2.61 detected in URP2R with an average of 9.85 per primer (Table 4).

### Genetic diversity analysis

Variations among and within the *Rosa* sp. populations were evaluated by molecular variance analysis (AMOVA) (Table 5). Based on combined markers data, the results revealed a higher variation within regions (86%), while 14% of the variation was recorded among the regions with a significant PhiPT value (PhiPT = 0.14, *p* = 0.001).

**Table 5:** Analysis of molecular variance (AMOVA) based on ISSR and DAMD data in *Rosa* sp. populations

Source	df	SS	MS	Est. Var.	Var
**Among Pops**	9	898.647	99.850	9.873	14%
**Within Pops**	29	1810.738	62.439	62.439	86%
Total	38	2709.385		72.312	100%
PhiPT = 0.137					
*P* = 0.001					

*Df* degree of freedom, *SS* sum of squares, *MS* mean of squares, *Est. Var* estimated variance components, *Var* total variance

Percentage of polymorphic loci (PPL) of Roses using the combined ISSR and DAMD markers (Table 6) spanned from 21.92% (Ait Touda) to 73.07% (Azrou). The Azrou region recorded the highest value of the observed number of alleles (Na), effective number of alleles (Ne), Shannon’s information index (I), and expected heterozygosity (He) with values of 1.49, 1.34, 0.33, and 0.21, respectively. On the other hand, Ait Touda registered the lowest values (0.59, 1.15, 0.13, and 0.09, respectively).

**Table 6:** Genetic variation among different regions of Moroccan wild rose populations as revealed through ISSR and DAMD analysis

Region	Na	Ne	I	He	PPL
Tamalout	0.929	1.268	0.229	0.155	40.08
Akka	0.795	1.213	0.182	0.125	30.06
Bouadel	1.111	1.262	0.241	0.157	49.48
Agoudim	0.948	1.260	0.225	0.151	41.13
Tirghist	0.923	1.259	0.224	0.151	39.67
Tounfite	0.998	1.280	0.243	0.163	44.68
Kela Mgona	1.159	1.311	0.283	0.187	54.49
Taoudaat	0.691	1.204	0.174	0.119	28.81
Azrou	1.486	1.336	0.330	0.211	73.07
Ait Touda	0.595	1.155	0.133	0.091	21.92
*Mean*	*0.963*	*1.255*	*0.226*	*0.151*	*42.34*

*Na* observed number of alleles, *Ne* effective number of alleles, *I* Shannon’s information index, *He* expected heterozygosity, *PPL* percentage of polymorphic loci

### Genetic distance

Table 7 revealed the genetic distance among 10 regions including 39 accessions of *Rosa* sp. based on the combined ISSR and DAMD matrix that ranged from a minimum of 0.05 to a maximum of 0.19. The highest genetic distance (0.19) was registered for the pair of regions such as Kela Mgona and Akka Ait Ouyed. However, the minimum genetic distance (0.05) was recorded for the pair regions of Bouadel and Agoudim.

**Table 7:** Pairwise Population Matrix of Nei genetic distance among 10 regions included 39 accessions of rose, calculated using ISSR and DAMD data

	Tamalout	Akka	Bouadel	Agoudim	Tirghist	Tounfite	K. Mgona	Taoudaat	Azrou	Ait touda
**Tamalout**	0.000									
**Akka**	0.132	0.000								
**Bouadel**	0.090	0.098	0.000							
**Agoudim**	0.083	0.102	0.050	0.000						
**Tirghist**	0.114	0.080	0.064	0.089	0.000					
**Tounfite**	0.099	0.117	0.056	0.080	0.093	0.000				
**Kela Mgona**	0.147	0.192	0.152	0.150	0.151	0.136	0.000			
**Taoudaat**	0.110	0.174	0.117	0.108	0.132	0.121	0.130	0.000		
**Azrou**	0.093	0.146	0.103	0.103	0.103	0.110	0.107	0.108	0.000	
**Ait touda**	0.099	0.174	0.109	0.104	0.125	0.124	0.177	0.111	0.102	0.000

### Cluster analysis

**Fig. 3:**
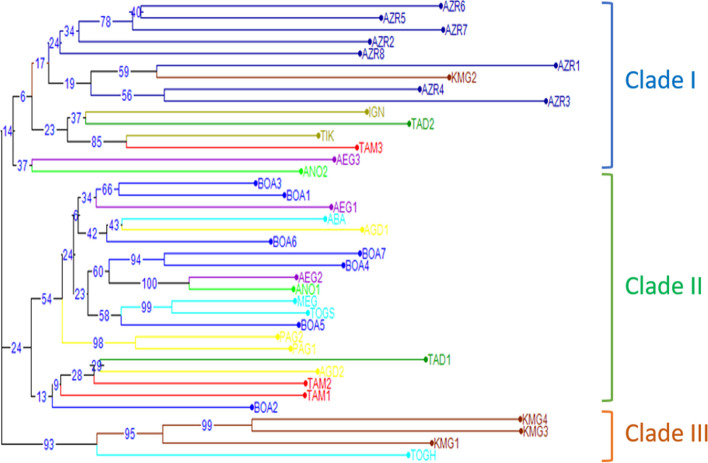
Dendrogram based on the dissimilarity index values of 39 rose accessions using ISSR and DAMD markers. Accessions are as indicated in Table 1. Bootstrap values (%, based on 1000 replications)

The dissimilarity index calculated with data from the combined matrix of ISSR and DAMD markers and used to build a Weighted Neighbor-Joining tree ranged from 0.26 to 0.84. At 70% of dissimilarity, the dendrogram divided the 39 accessions into three major clusters (Fig. 3). The first cluster consisted of 15 wild accessions from Azrou (8 accessions), Ait Touda (2 accessions), and Kela Mgona, Taoudaat, Akka Ait Ouyed, Tirghist, and Tamalout (1 accession). The second cluster comprised 20 wild accessions springing from Bouadel (7 accessions), Agoudim (4 accessions), Tirghist (2 accessions), Tounfite (3 accessions), Tamalout (2 accessions), and Taoudaat and Akka Ait Ouyed (1 accession). Four accessions were classified in the third cluster and comprised 4 hybrid accessions from Kela Mgona (3 accessions) and Tounfite (1 accession).

### Principal component and population structure analyses

**Fig. 4:**
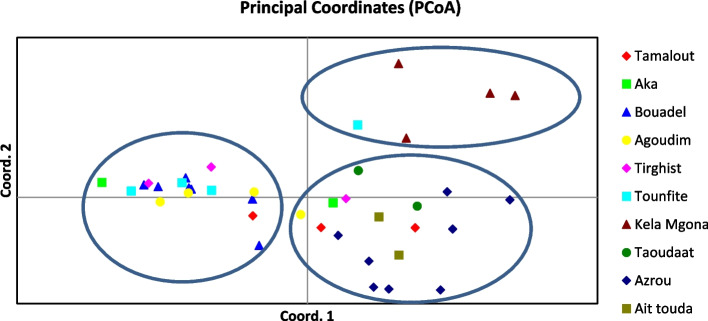
Principal coordinate analyses (PCoA) for 39 Moroccan *Rosa* sp. accessions based on ISSR and DAMD data

The principal component analysis (PCoA) made it possible to graphically represent the relationship between the ISSR and DAMD markers, and the different accessions (Fig. 4). Derived on the basis of ISSR and DAMD markers data, the first three axes describe a total variation of 26.26%. The first axis (PC1) expressed the largest variation with 12.78% and, the second (PC2) and the third (PC3) accounted for 7.33 and 6.15%, respectively. PCoA showed a significant overlap between accessions, except for 4 accessions from Kela Mgona and 1 accession from Tounfite which was gathered far from other regions.

**Fig. 5:**
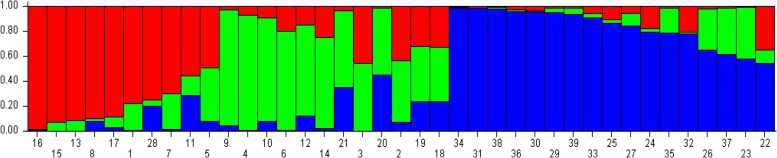
Population structure of 39 rose accessions based on ISSR and DAMD data

Using the structure software, we can determine the best value of *k* in order to characterize the genetic structure of 39 accessions. The program detected the highest value of delta k obtained from the structure harvester, *k*=3. Based on the best *k*=3, each assessed accession is often positioned into three different subgroups (Fig. 5). Subgroup 1 consisted of all accessions from Kela Mgona, 4 accessions from Azrou, 1 accession from Tounfite and Akka Ait Ouyed, whereas all accessions from Ait Touda and Taoudaat, 4 accessions from Azrou, 2 accessions from Tamalout and 1 accession from Agoudim and Tirghist were grouped into subgroup 2. All accessions from Bouadel, Tounfite (3), Agoudim (3), Tirghist (2), Akka Ait Ouyed, and Tamalout (1) were allocated to subgroup 3.

## Discussion

The classical phylogenetic approach relies on the morphological characteristics of an organism. *Rosa* also have a wide and overlapping range of morphological variations which are influenced by the environment. Classification based on morphology alone is not adequate [36]. Therefore, for these reasons, several molecular markers have been applied in the genetic studies of roses, notably AFLP, RAPD, ISSR, SSR, SCoT, URP, CDDP, and DAMD. The choice of the molecular marker is an important step for researchers and especially geneticists and breeders. Our study analyzed the genetic diversity of the Moroccan rose population, using two PCRs marker molecular ISSR and DAMD.

ISSR and DAMD markers produced high levels of polymorphism, such as the average number of scored bands, polymorphism rates, and PIC were slightly higher with the ISSR primers (27.3, 99.08%, and 0.34, respectively) compared to those of the DAMD primers (25.37, 95.83%, and 0.31, respectively). The high rate of polymorphism revealed by both markers may have been due to the difference in ploidy level, mutations, recombination, natural hybridization, or random segregation of heterozygous chromosomes in the process of meiosis [37]. The polymorphism rate obtained using ISSR and DAMD primers during this study was close to those observed with 47 genotypes of *Rosa* in Iran (90%) by [15]; this rate was higher than those reported by [13] using ISSR on 7 species of *Rosa* and by [23] on 23 genotypes of *Rosa canina*, reaching 75% and 77%, respectively. The mean of polymorphic band obtained in this study using ISSR and DAMD were 27.4 and 25, respectively, and this is much higher than the results reported by [23] using ISSR with an average of 3 and by [38] using DAMD with a mean of 5.87; this is probably due to the fact that we used polyacrylamide gel which revels more band than agarose gel.

In our study, the efficiency of markers was determined by effective multiplex ratio (EMR), marker index (MI), and resolving power (Rp). The values detected for EMR, MI and Rp (8.95, 3.05, and 11.95, respectively) using the ISSR primers are higher than those obtained by DAMD primers (6.68, 2.11, and 9.85, respectively). These results show that the ISSR marker is very efficient compared to the DAMD marker. In accordance with [39], the results revealed that ISSR profiling is an effective tool for assessing genetic diversity.

According to the AMOVA analysis, 86% of genetic variations within regions were shown by the combined ISSR and DAMD markers, indicating that the variation within accessions was higher (86%) than among them (14%). Many researches obtained similar results. Indeed, [40] using URP and SCoT markers on 40 Damask rose genotypes and [41] using 12 pairs of universal cpDNA primers on 12 wild populations revealed within population values of 93% and 77%, respectively, while they obtained 7% and 23% among population. The authors reported that the genetic variation within the collected populations was higher than the variation among them. This indicates that the genetic variation in wild rose mainly occurred within populations.

Maximum values of indices in relation to genetic diversity (Na, Ne, I, He, and PPL) were reported for the Azrou region using combined data. This result indicates that this region could be an important source of diversity for breeding projects which can benefit from new alleles and genes of interest.

Variable genetic distances among rose populations were registered in this study. Results revealed that regions with the lowest distance (Bouadel and Agoudim: 0.05) are found in adjacent geographic regions. This shows a possible existence of seed movement among these areas; unlike populations Kela Mgona (included hybrid accessions) and Akka Ait Ouyed, which exhibited large genetic distances (0.19).

The dissimilarity index calculated with data from the combined matrix of ISSR and DAMD markers ranged from 0.265 to 0.84 with a similarity of 30%. Various studies revealed a high similarity value compared to our findings [42]. using RAPD and SSR markers on 71 Indian local varieties of roses, [15] using ISSR markers, [38] using SCoT, CCDP, and DAMD primers, and [43] using SSR markers detected similarity values with 63%, 52%, 56%, and 67%, respectively. This proved a high genetic variability of roses in Morocco.

Three clusters were recorded in this study. In cluster I, there is an overlap between the regions Ait Touda, Taoudaat, Tamalout, Tirghist, and Akka Ait Ouyed. On the other hand, the accessions of the Azrou region are grouped together in a single sub-clade with the integration of KMG2 accession from Kela Mgona. As a result, the region of Azrou where the endemic species of Morocco *Rosa mesatlantica* [8] was included is a genetically conserved area that can be considered as a source of variability. Moreover, this indicates that this region could be an important source of diversity. This result leads us to the exploitation of these wild resources and to carry out more research on them. In cluster II, there is also an overlap between the seven regions. Therefore, there is no genetic conservation within these regions. The four hybrids of Kela Mgona and Tounfite are grouped together in the third cluster. This is normal due to the morphological and genetic differences between domesticated and wild rose species. The results of clustering showed that geographical origin was not the main factor causing the genetic differentiation among the wild rose populations.

In the present studies, the results of PCoA analysis were consistent with those of the cluster analysis. The accessions were well distributed in the biplot, which could be due to the relevance of primers used and the wide variety between accessions. The PCoA revealed that Bouadel, Tirghist, Tounfite, Agoudim, and Akka Ait Ouyed regions are very close to each other, which indicates that there is a high genetic flow among them, while this did not follow their origins. The results of PCoA indicated that the wild rose populations were expanded. There may be multiple refuges for wild rose, such as the Azrou and Bouadel populations.

Roses usually cross-pollinate and are self-incompatible which makes them more genetically diverse between and within populations [44–46]. analyzed 55 genotypes of roses in Brandenburg (Germany) which were grouped in twelve clusters, whereas the high genetic variation was observed within *Rosa canina* populations. These authors stated that this variation was due to the polyploidy, outcrossing, and seed dispersal systems. That is in accordance with our study which also showed that most of the species present in Morocco belong to the caninae section [8].

Using the structure harvester software, the best K were identified. The best level of genetic structure in the combined data system was K=3. In this study, high gene flow was detected among rose accessions. This is probably because of plant transmissions by humans or genetic flow and displacement by natural factors [47]. This genetic variation should be considered as a valuable genetic source for breeding programs.

## Conclusion

This study showed the genetic diversity of Moroccan wild rose accessions using ISSR and DAMD markers. These markers detected a high level of polymorphism in these accessions. The PIC average values for both markers were 0.34 and 0.31, respectively, which indicates the efficiency of used markers in revealing polymorphism among wild rose accessions. Furthermore, the findings confirmed the efficiency of combined data in estimating the genetic diversity among the populations. The study of genetic diversity is a very important and crucial step that can help us to think of how to use this high level of variation in the breeding program and also how to manage and conserve those wild genetic resources. This study shows also that it is important to exploit wild resources for the genetic improvement of hybrid roses, especially *Rosa damascena* in Morocco, especially since this country is known as one of the main producers of these roses in the world.

## Data Availability

All data generated or analyzed during this study are included in this article.

## Abbreviations

PCR: Polymerase chain reaction
RAPD: Random amplified polymorphic DNA
SSR: Simple sequence repeat
RFLP: Restriction fragment length polymorphism
SCoT: Start codon targeted
URP: Universal rice primers
CCDP: Conserved DNA-derived polymorphism
iMEC: Marker efficiency calculator
